# Common features of F-18 FDG PET/CT findings in Scrub Typhus: prospective study before and after antibiotics therapy

**DOI:** 10.1038/s41598-019-51964-6

**Published:** 2019-10-28

**Authors:** Joo-Hee Hwang, Yeon-Hee Han, Seung Hee Choi, Mir Jeon, Suhyun Kim, Yeon-Joon Kim, Chang-Seop Lee, Seok Tae Lim

**Affiliations:** 1 Department of Internal Medicine, Chonbuk National University Medical School, Jeonju, Republic of Korea; 2 Department of Nuclear Medicine, Chonbuk National University Medical School, Jeonju, Republic of Korea; 3 Research Institute of Clinical Medicine of Chonbuk National University-Biomedical Research Institute of Chonbuk National University Hospital, Jeonju, Republic of Korea; 4 Cyclotron Research Center, Molecular Imaging and Therapeutic Medicine Research Center, Chonbuk National University Medical School and Hospital, Jeonju, Republic of Korea; 5 Department of Industrial Design, Chonbuk National University, Jeonju, Republic of Korea

**Keywords:** Clinical microbiology, Bacterial infection

## Abstract

Scrub typhus is an acute febrile illness caused by obligate intracellular organism *Orientia tsutsugamushi*. While there have been many reports on the evaluation of disease activity and infectious diseases using F-18 fluorodeoxyglucose (FDG) positron emission tomography/computed tomography (PET/CT), the clinical value of FDG PET/CT in scrub typhus has not been fully investigated. We enrolled 17 patients who were 18 years of age or older and clinically suspected of having scrub typhus with eschar. Clinical assessments, blood samples, and FDG PET/CT images were obtained at enrolment and again after 3 weeks. The median age of the patients was 65 years; 9 (52.9%) patients were male. On initial FDG PET/CT, the eschars showed markedly increased FDG uptake on PET imaging that improved after treatment. Generalized lymphadenopathy and splenomegaly with high FDG uptake were observed in all patients. On follow-up FDG PET/CT after appropriate therapy, FDG uptake and sizes of eschar, lymph nodes, and spleen were markedly decreased. As far as we are aware, this is the first investigation with multiple patients of FDG PET/CT in scrub typhus and the demonstration of clinical utility. FDG PET/CT imaging of scrub typhus could provide useful information about the clinical features before and after antibiotic treatment.

## Introduction

Scrub typhus is an acute febrile illness caused by the obligate intracellular organism *Orientia tsutsugamushi*, which is transmitted by the bite of infected larvae of the trombiculid mite (chigger)^1^. An eschar, a typical sign of scrub typhus, is formed at the site of the chigger bite in about 10–92% of scrub typhus patients^2,3^. Within the eschar site, *O. tsutsugamushi* invades and replicates inside activated monocytes/macrophages and dermal dendritic cells^4–6^. And then, activated dendritic cells actively migrate to regional lymph nodes via lymphatic vessels^7,8^. This is followed by bacteremia, where *O. tsutsugamushi* invades the endothelial cells, leading to focal or disseminated multi-organ vasculitis^9,10^. In addition, a large number of *O. tsutsugamushi* are distributed in the reticuloendothelial system, such as liver, spleen, lymph node, and bone marrow^11^.

The diagnosis of scrub typhus is based on a history of exposure, clinical features, and results of serologic testing. While there have been some studies on the computed tomography (CT) findings in scrub typhus, the radiologic findings are varied and nonspecific^12^. Moreover, definite F-18 fluorodeoxyglucose (FDG) positron emission tomography/computed tomography (PET/CT) imaging findings in scrub typhus have not been demonstrated. To date, only 3 cases have been reported on FDG PET/CT in scrub typhus, and 2 of them had underlying malignancies^13–15^.

FDG PET/CT is commonly used to detect or evaluate the tumorous condition in the oncologic field. Furthermore, the usefulness of imaging has been shown for infectious disease due to the high expression of glucose transporters of neutrophils and the monocyte/macrophage^16–19^. Since activated macrophages, which are known to express high levels of glucose transporters, have a critical role in the pathogenesis of scrub typhus, FDG PET/CT might provide useful information for diagnosis. While there have been many reports on the evaluation of disease activity and extent of bacterial, viral, or mycobacterial infection using FDG PET/CT, the clinical utility of FDG PET/CT in scrub typhus has not been fully investigated. Therefore, we carried out a prospective study to describe the FDG PET/CT findings and its clinical implications in scrub typhus patients.

## Results

**Figure 1:**
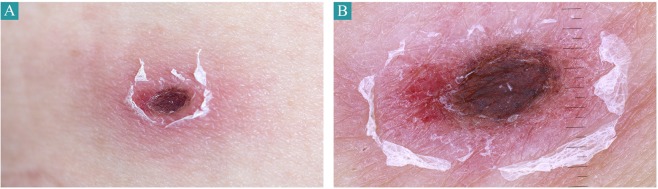
Typical eschar. (**A**) Clinical photograph of eschar showing a 1.4 cm × 1.7 cm sized, black crust surrounded by erythematous rim and peripheral collarette of white scale. (**B**) The clinical appearance of the eschar is well evaluated by dermoscopy.

Over the study period, 18 scrub typhus patients with typical eschar were enrolled (Fig. 1). One patient was lost to follow-up. Finally, a total of 17 patients were included in the study. The median age of the patients was 65 years; 9 (52.9%) were male. The majority of patients were engaged in agricultural activities (12, 70.6%) and presented with a skin rash (10, 58.8%). Ten (58.8%) had a headache, and 6 (35.3%) developed gastrointestinal symptoms such as dyspepsia, nausea/vomiting, or abdominal pain. In laboratory findings, all patients showed mild to moderate elevation of liver function tests despite no underlying liver diseases, and most patients also had thrombocytopenia on admission. However, the abnormal laboratory findings had normalized on the second visit. In addition, the most commonly identified genotypes of *O. tsutsugamushi* were the Boryong strain (15, 88.2%), which is the predominant strain throughout South Korea. All patients were successfully treated with a 7-day course of oral doxycycline 200 mg/day. The demographic and clinical characteristics of the enrolled patients are summarized in Table 1.

**Table 1:** Demographic, Clinical Characteristics, and Laboratory Findings on Admission of the Scrub Typhus Patients.

Characteristics	Scrub typhus (n = 17)
Demographic data, median (IQR)	
Age (years)	65 (57.5–78.0)
Male, no. (%)	9 (52.9)
Agricultural activities, no. (%)	12 (70.6)
Duration of illness before admission (days)	6.3 (3.0–9.0)
Hospitalization days	5.4 (4.0–5.5)
Comorbidities, no. (%)	
Diabetes mellitus	2 (11.8)
COPD/Asthma	1 (5.9)
Clinical signs & symptoms, no. (%)	
Headache	10 (58.8)
Dyspepsia	6 (35.3)
Nausea/Vomiting	5 (29.4)
Abdominal pain	6 (35.3)
Fever	17 (100.0)
Rash	10 (58.8)
Eschar	17 (100.0)
Laboratory values, median (IQR)	
WBC count, x1,000/mm^3^	7.7 (3.9–10.8)
Platelet count, x1,000/mm^3^	139.0 (96.0–176.0)
PT, INR	1.1 (1.0–1.2)
Total bilirubin, mg/dL	0.74 (0.44–0.85)
Albumin, g/dL	3.6 (3.3–4.0)
AST, IU/L	124.4 (76.5–147.5)
ALT, IU/L	118.3 (54.0–130.5)
ALP, IU/L	121.5 (68.5–188.5)
Creatinine, mg/dL	0.9 (0.7–1.0)
hs-CRP, mg/dL	107.1 (60.2–148.9)
Genotype, no. (%)	
Boryong Strain	15 (88.2)
Karp strain	1 (5.9)
Kawasaki strain	1 (5.9)
Treatment, no. (%)	
Doxycycline	17 (100.0)

Notes: Data are presented as median (Interquartile range) or number (percentage). Abbreviations: IQR, Interquartile range; COPD, Chronic obstructive pulmonary disease; WBC, white blood cell; PT, prothrombin time; AST, aspartate aminotransferase; ALT, alanine aminotransferase; ALP, alkaline phosphatase; CRP, C-reactive protein.

**Figure 2:**
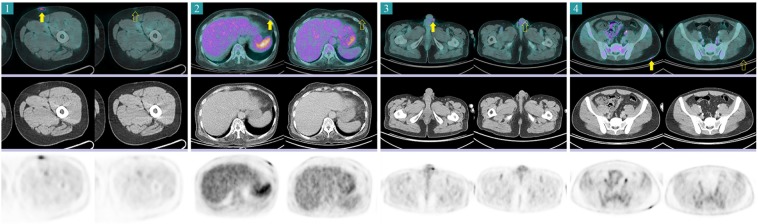
Eschar lesions from 4 patients. Before antibiotic treatment (each left columns), eschar lesions appeared as hypermetabolic skin thickenings. Skin thickness and FDG uptake were markedly decreased after treatment (each right columns). The yellow arrows indicate the pretreatment eschar lesions, and the yellow empty arrows indicate the same sites after treatments.

**Figure 3:**
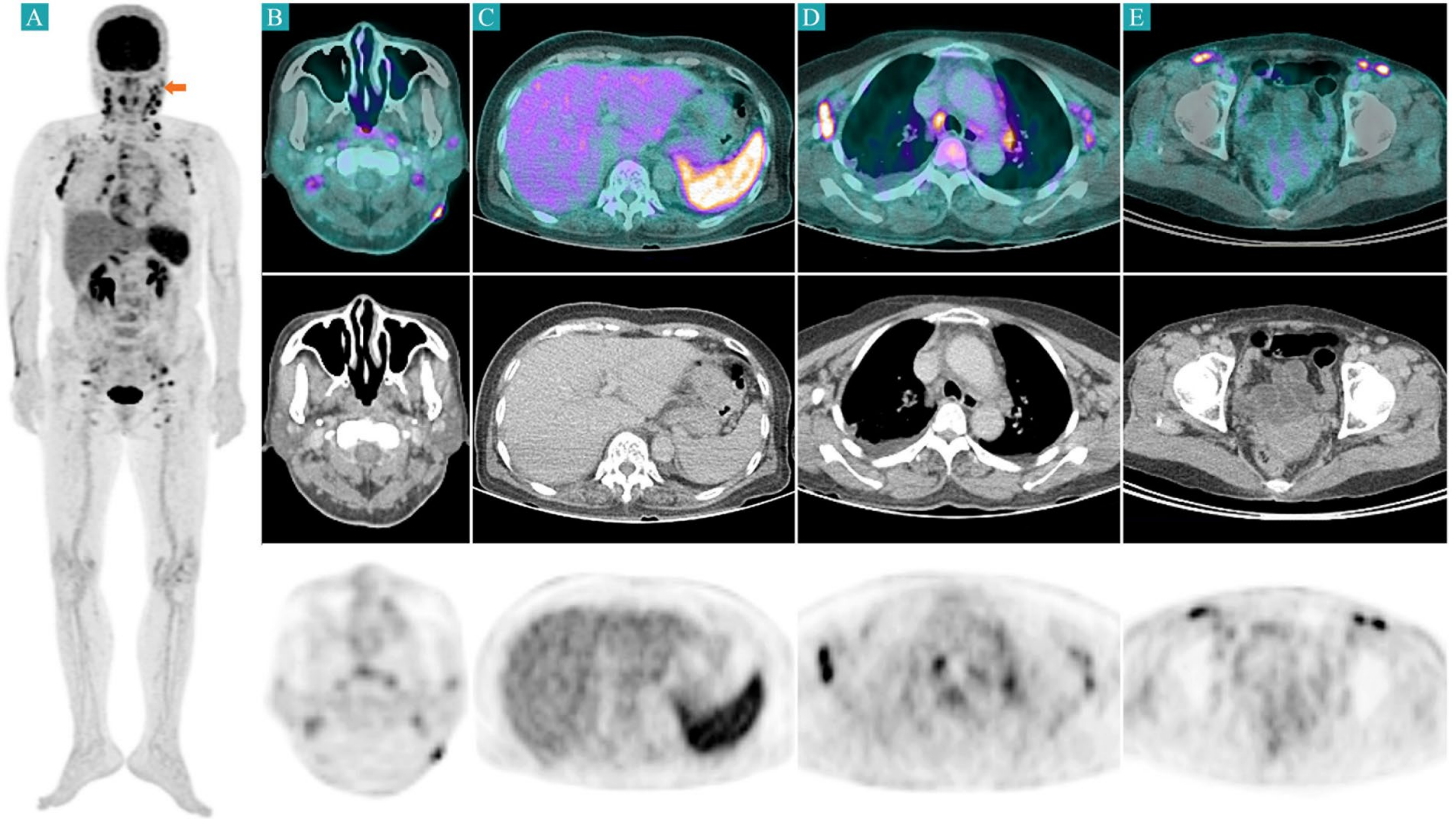
Initial FDG PET/CT image of a 71-year-old female patient with scrub typhus. Maximum intensity projection image shows generalized lymphadenopathy from bilateral cervical to inguinal areas and hypermetabolism in the spleen (**A**). Focally increased FDG uptake is demonstrated on the eschar lesion in the left posterior neck (**B**). Splenomegaly with hypermetabolism and generalized lymphadenopathy in the lymph node-bearing sites were also demonstrated (**C–E**). The red arrow indicates the eschar lesion.

**Figure 4:**
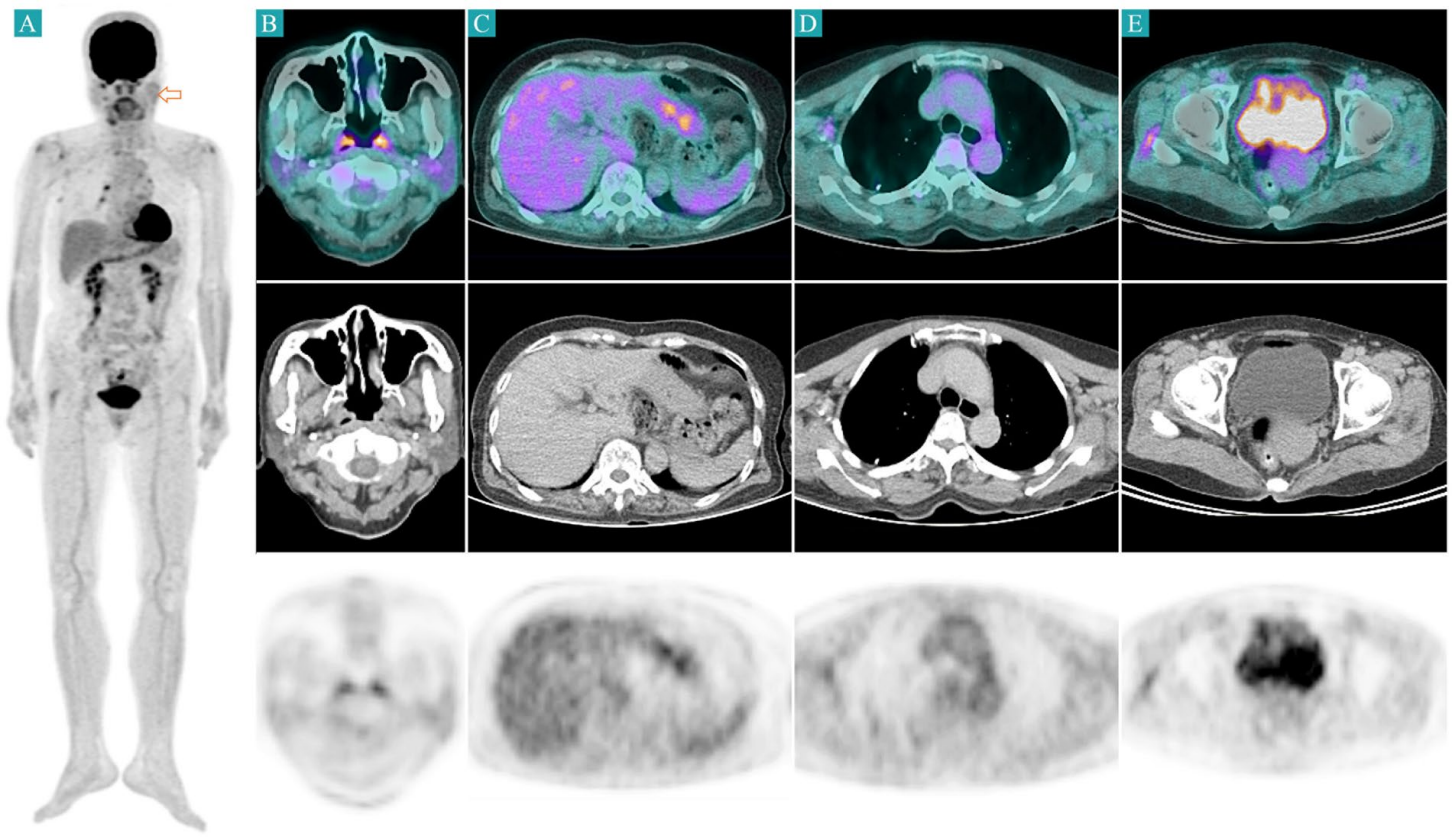
Follow-up FDG PET/CT image of the same patient in Fig. 3. FDG uptake and the size of eschar, lymph nodes, and spleen are markedly decreased after proper antibiotic therapy (**A–E**). The red empty arrow indicates the eschar lesion.

On the initial FDG PET/CT, eschar lesions of all patients showed high FDG uptake (Fig. 2). Generalized lymphadenopathy and splenomegaly with high FDG uptake were also found in all patients. Four of the 17 patients had a pleural effusion. The initial FDG PET/CT image of a representative case is illustrated in Fig. 3. On the follow-up FDG PET/CT, FDG uptake and the sizes of eschar, lymph nodes, and spleen were markedly decreased (Fig. 4). In transverse CT analysis of initial and follow-up FDG PET/CT, the maximal short axis of lymph node, longest diameter of the spleen, and longest anterior-posterior diameter of the liver showed a significant decrease after proper antibiotic therapy. On semi-quantitative analysis of FDG PET/CT, SUVmax of eschar, lymph node, and spleen was markedly decreased on follow-up FDG PET/CT image. SUVmean of spleen showed marginal significance with a *P*-value of 0.066. However, SUVmax and SUVmean of the liver showed no significant interval change between initial and follow-up FDG PET/CT. Details are summarized in Table 2.

**Table 2:** Variable Parameters in Initial and Follow-up FDG PET/CT.

Parameters	Initial FDG PET/CT median (IQR)	Follow-up FDG PET/CT median (IQR)	*P*-value†
Maximal short axis of lymph node (cm)	1.1 (0.9–1.2)	0.6 (0.5–0.8)	0.000
Maximal length of spleen (cm)	11.2 (10.4–12.8)	9.7 (8.7–10.7)	0.000
Maximal A-P length of liver (cm)	15.6 (14.7–16.2)	14.6 (13.8–15.8)	0.000
SUVmax of eschar lesion	3.31 (2.38–4.43)	1.41 (1.15–1.68)	0.000
SUVmax of lymph node	10.36 (7.80–12.81)	2.23 (2.03–3.36)	0.000
SUVmax of spleen	6.01 (5.06–6.34)	3.10 (2.89–3.31)	0.000
SUVmean of spleen	3.74 (3.01–4.22)	1.88 (1.82–1.95)	0.004
SUVmax of liver	3.72 (3.32–4.30)	3.92 (3.56–4.43)	0.619
SUVmean of liver	2.20 (1.91–2.45)	2.39 (2.13–2.57)	0.149

Notes: ^†^Analyzed by Wilcoxon signed rank sum test. Abbreviations: IQR, interquartile range; A-P, anterior posterior.

## Discussion

Based on our FDG PET/CT analysis, high FDG uptake of eschar, lymph nodes, and spleen and increased sizes of lymph nodes, spleen, and liver were observed in scrub typhus patients.

First, the eschars showed dramatically increased FDG uptake on PET imaging and were improved after treatment in all patients. Eschar is a well-known portal of entry for *O. tsutsugamushi*, which replicates in monocytes/macrophages and dendritic cells. For this reason, many activated monocytes/macrophages accumulate in the eschar and cause high FDG uptake in the lesion. Even though an eschar is a pathognomonic feature of scrub typhus, the incidence of eschar is highly variable, with higher (>80%) values being reported from South Korea, Japan, and China and lower (<30%) values from India, Thailand, and Sri Lanka. Therefore, FDG PET/CT is to be as a promising imaging tool providing the common imaging features in eschar-negative scrub typhus.

Second interesting FDG PET/CT findings were generalized lymph node enlargement and increased FDG uptake in lymph nodes. In line with other systemic inflammatory diseases, generalized and symmetrical lymphadenopathy was observed in our scrub typhus patients. The size of lymph nodes was mild to moderately increased, and FDG uptake was moderate to intense, which is similar to that of other inflammatory lymph nodes. Although palpable lymph node enlargement is a relatively well-known sign in scrub typhus, the reported degree varies from 12% to 85%^20,21^. In this study, lymph node enlargement was observed in all patients. This finding suggests that lymph node enlargement accompanies and is essential in scrub typhus.

Third, distinctive FDG PET/CT findings were hepatosplenomegaly and hypermetabolism of spleen. In prior studies, abnormal liver function in scrub typhus was reported in around 90% of patients due to histopathologic changes such as swollen hepatocytes and small lymphocyte aggregation in sinusoids or with granulomatous changes. Before antibiotic treatment, the size of the liver was markedly increased and was within the normal range after appropriate treatment. However, FDG uptake was not significantly increased in the liver. Though the reason for isometabolism in the liver is not yet fully understood, hepatosplenomegaly combined with hypermetabolism in the spleen is frequently encountered in systemic inflammatory disease. Hypermetabolic splenomegaly was seen in all patients of this study and could be a clinically useful predictor of scrub typhus. Furthermore, FDG PET/CT is a sensitive modality for evaluating the effects of antibiotic therapy. Even though *O. tsutsugamushi* is known to involve the kidneys, lungs, and brain, we could not find any abnormal metabolism in those organs on FDG PET/CT images.

Since FDG PET/CT has been widely used in variable clinical fields, the evaluation of several infectious and inflammatory disorders using this imaging modality has been also attempted and provided useful clinical evidence^18,22^. Because inflammatory cells use glucose as their energy source like malignant cells, FDG accumulation at the sites of infection has been considered as a pitfall for interpretation of malignancy^18,23,24^. However, this phenomenon could be applied to examine and manage patients with infectious and inflammatory diseases. Out of variable biological mechanisms of FDG-avidity in activated inflammatory cells such as macrophage, neutrophil, and lymphocyte, overexpression of glucose transporter 1 is considered to contribute mostly to the phenomenon^18,25^.

We are aware of the limitations of this study. As it was a single-center study including a small number of patients, the results should be interpreted with caution and should not be generalized to all patients. In spite of this limitation, all patients of this study had very similar FDG PET/CT findings, and we believe that the results will be the same in other scrub typhus patients. By carrying out FDG PET/CT in scrub typhus patients, we showed that *O. tsutsugamushi* spread to lymph nodes in multiple sites via lymphatic vessels and involved multiple organs. In addition, an awareness of the related findings at imaging, especially at FDG PET/CT, may provide clues for diagnosis of scrub typhus in non-endemic areas or eschar-negative scrub typhus.

To our knowledge, this study is the first investigation with multiple patients of FDG PET/CT in scrub typhus and the demonstration of clinical utility. We also showed the typical findings of FDG PET/CT such as high FDG uptake of eschar, lymph nodes, and spleen and increased sizes of lymph nodes, spleen, and liver. Therefore, FDG PET/CT imaging of scrub typhus could provide valuable information about the clinical features before and after antibiotic treatments.

## Methods

### Patients and data collection

A single-center prospective study was conducted in a 1,200-bed tertiary hospital between September 2017 and December 2018. Patients ≥18 years of age who were clinically suspected of having scrub typhus with eschar were eligible. Patients with malignancies were excluded. Clinical assessments were performed at enrolment and 21 (±3) days later. The initial blood specimens and FDG PET/CT images were obtained prior to or within 2 hours of the start of antibiotics, and follow-up blood specimens and FDG PET/CT were acquired 3 weeks after appropriate treatments. Scrub typhus was confirmed by an increase in the indirect immunofluorescence assay (IFA) IgM titer ≥1:160 against *O. tsutsugamushi*, an increase in the IFA IgG titer against *O. tsutsugamushi* to ≥1:256, or a ≥4-fold increase in the IFA titer *O. tsutsugamushi*, or when a positive reaction was observed in a nested polymerase chain reaction (PCR) targeting the 56-kDa gene of *O. tsutsugamushi*. Demographic and clinical information was collected from electronic medical records, including age, sex, comorbidities, signs and symptoms, and laboratory data.

### Genotyping by DNA amplification and sequencing

Peripheral blood mononuclear cells collected from acute-phase blood samples of scrub typhus patients were purified using a QIAamp DNA Blood Mini Kit (QIAGEN GmbH, Hilden, Germany) according to the manufacturer’s protocol. Nested PCR was performed. Primers 34 (forward, 5′-TCA AGC TTA TTG CTA GTG CAA TGT CTGC-3′; the 56-kDa gene based on the Gilliam strain) and 55 (5′-AGG GAT CCC TGC TGC TGT GCT TGC TGCG-3′) were used in the first PCR. Nested PCR primers 10 (5-GAT CAA GCT TCC TCA GCC TAC TAT AAT GCC-3) and 11 (5-CTA GGG ATC CCG ACA GAT GCA CTA TTA GGC-3) were used in the second PCR amplification to generate a 483 bp fragment. Nested PCR was performed as described previously by Kim *et al*.^26^. The amplified PCR products were confirmed by 1.2% agarose gel electrophoresis, purified using a QIAquick gel extraction kit (QIAGEN), and sent to COSMO Genetech (Seoul, Korea) for sequencing.

### F-18 FDG PET/CT acquisition

All patients fasted for at least 6 hours prior to the intravenous injection of [^18^F]FDG, and the blood glucose level of all patients was below 140 mg/dL. Approximately 5.5 MBq (0.15 mCi) of [^18^F]FDG per kilogram of body weight was administered intravenously. Scanning was performed 60 minutes after [^18^F]FDG administration. Whole body images were obtained using a Biograph TruePoint 40 PET/CT scanner (Siemens Medical Solutions, Knoxville, TN, USA). A low-dose CT scan was obtained first for attenuation correction by a continuous spiral technique (120 kVp, 45 mA, 0.5 s rotation time). A PET scan was then acquired in 3-dimensional mode at 2 minutes per bed position. After the acquisition of PET data, the patient underwent a diagnostic CT scan with intravenous contrast (120 kVp, 200 mA, 0.5 s rotation time). The obtained PET data were reconstructed iteratively using an ordered-subset expectation maximization algorithm, and the initial CT data were used for attenuation correction.

### Image interpretation

Two experienced nuclear medicine physicians reviewed the initial and follow-up FDG PET/CT images on a workstation (Syngo MI applications, Flexible Display 7.0.7.7; Siemens Medical Solutions, Erlangen, Germany). We measured maximal short axis of the lymph node, longest diameter of the spleen, and longest anterior-posterior diameter of the liver on the transverse CT plane of FDG PET/CT. We also measured metabolic parameters of maximum standardized uptake value (SUVmax) of eschar, lymph node, liver, spleen and mean standardized uptake value (SUVmean) of liver and spleen. A volume of interest (VOI) was carefully drawn slightly larger than the target site in the axial, coronal, and sagittal planes. SUVmax, defined as the maximum SUV within the VOI, was calculated as follows: SUVmax = concentration of highest tumor activity in the VOI (MBq/mL) × total body weight (kg)/injected radioactivity (g/MBq). SUVmean was measured in a 3-cm-diameter VOI in the posterior portions of the liver and the spleen.

### Statistical analysis

Statistical analysis was performed using SPSS software (IBM; version 23, USA), and *P-*values less than 0.05 were considered statistically significant. Comparison between variable parameters in initial and follow-up FDG PET/CT was performed by Wilcoxon signed rank test, which is the nonparametric test of the paired t-test.

### Ethical statement

This study was conducted in accordance with Good Clinical Practice Guidelines and the Declaration of Helsinki. The study was approved by the institutional review board (IRB) of Chonbuk National University Hospital, and all patients provided written informed consent (IRB Registration Number 2017-08-033).

## Data Availability

Datasets generated during and/or analyzed during the current study are not publicly available but are available from the corresponding author on reasonable request.
